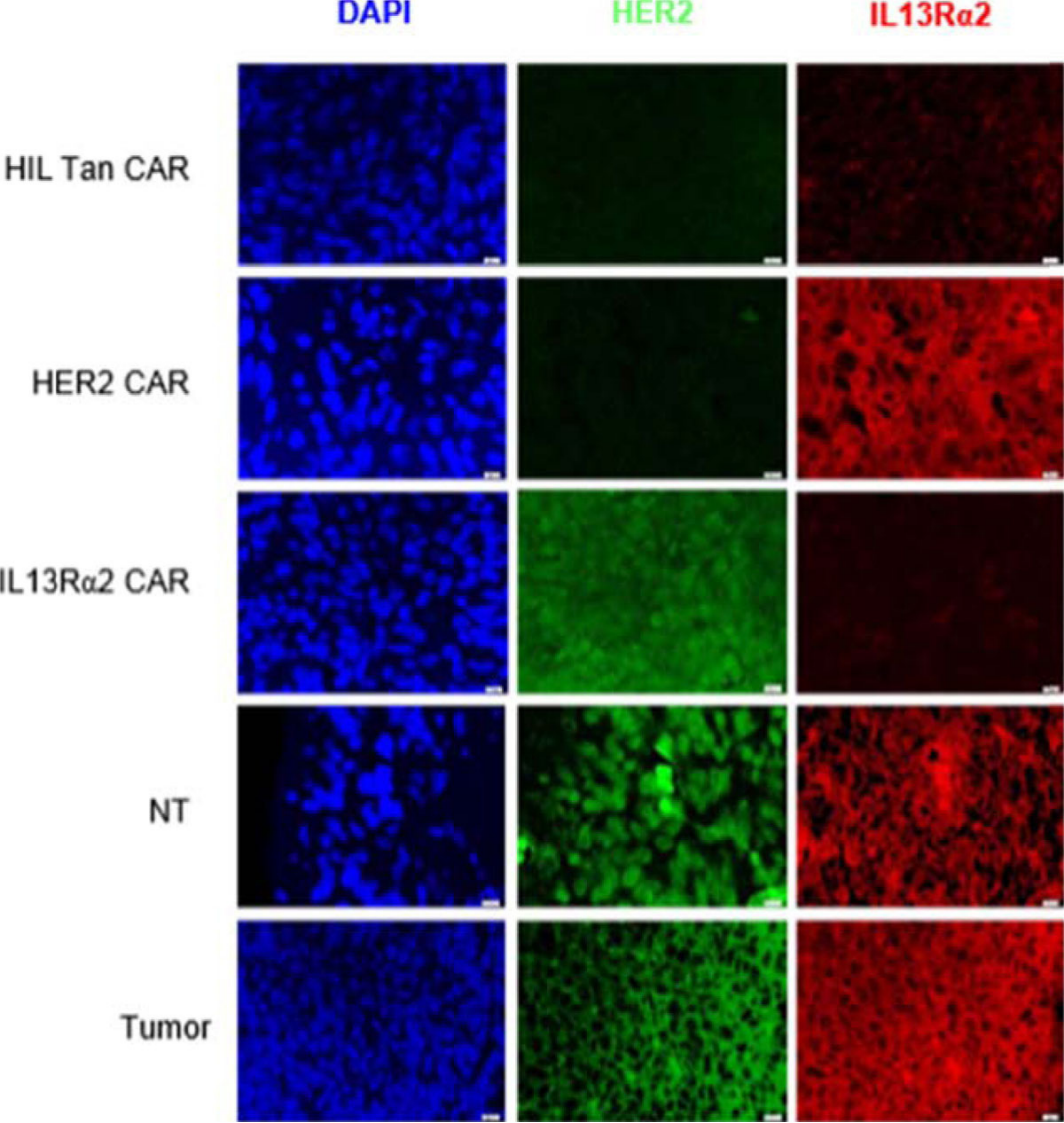# A bispecific chimeric antigen receptor molecule enhances T cell activation through dual immunological synapse formation and offsets antigen escape in glioblastoma

**DOI:** 10.1186/2051-1426-3-S2-O3

**Published:** 2015-11-04

**Authors:** Meenakshi Hegde, Zakaria Grada, Antonella Pignata, Amanda Wakefield, Kristen Fousek, Kevin Bielamowicz, Kevin Chow, Vita Brawley, Tiara Byrd, Stephen Gottschalk, Malini Mukherjee, Winfried S Wels, Matthew Baker, Giapietro Dotti, Jordan Orange, Nabil Ahmed

**Affiliations:** 1 Baylor College of Medicine, Houston, TX, USA; 2 Georg-Speyer-Haus, Institute for Tumor Biology and Experimental Therapy, Frankfurt, Germany

## Background

Antigen escape tumor cell variants prevail in tumors recurring after treatment with chimeric antigen receptor (CAR) T cells with a single specificity. Recurrent tumors preserve alternative non-targeted tumor associated antigens.

## Hypothesis

A bispecific CAR will mitigate antigen escape enhancing the antitumor activity of T cells.

## Methods and results

HER2 and IL13Rα2 are currently targeted in Phase I glioblastoma (GBM) trials using CAR T cells. We created a bispecific CAR molecule with a HER2-specific scFv joined in tandem to an IL13Rα2-binding moiety in the CAR exodomain (Tandem CAR) and a CD28.ζ signaling endodomain. We used computational modeling to interrogate this design. GBM patients' Tandem CAR T cells showed distinct binding to soluble HER2 and IL13Rα2 and killed primary autologous GBM cells. Three-dimensional reconstitution and quantification of confocal images of the Tandem CAR T cell/tumor interface revealed enhanced bifunctional immunological synapses compared to conventional CARs. Further, Tandem CAR T cells exhibited significantly enhanced inexhaustible activation dynamics when compared to conventional HER2 or IL13Rα2 CAR T cells and better controlled established GBM in an orthotopic murine model by offsetting both HER2 and IL13Rα2 escape.

## Conclusion

Tandem chimeric antigen receptors enhance T cell activation and mitigate antigen escape through bifunctional immunological synapse formation in GBM.

**Figure 1:**
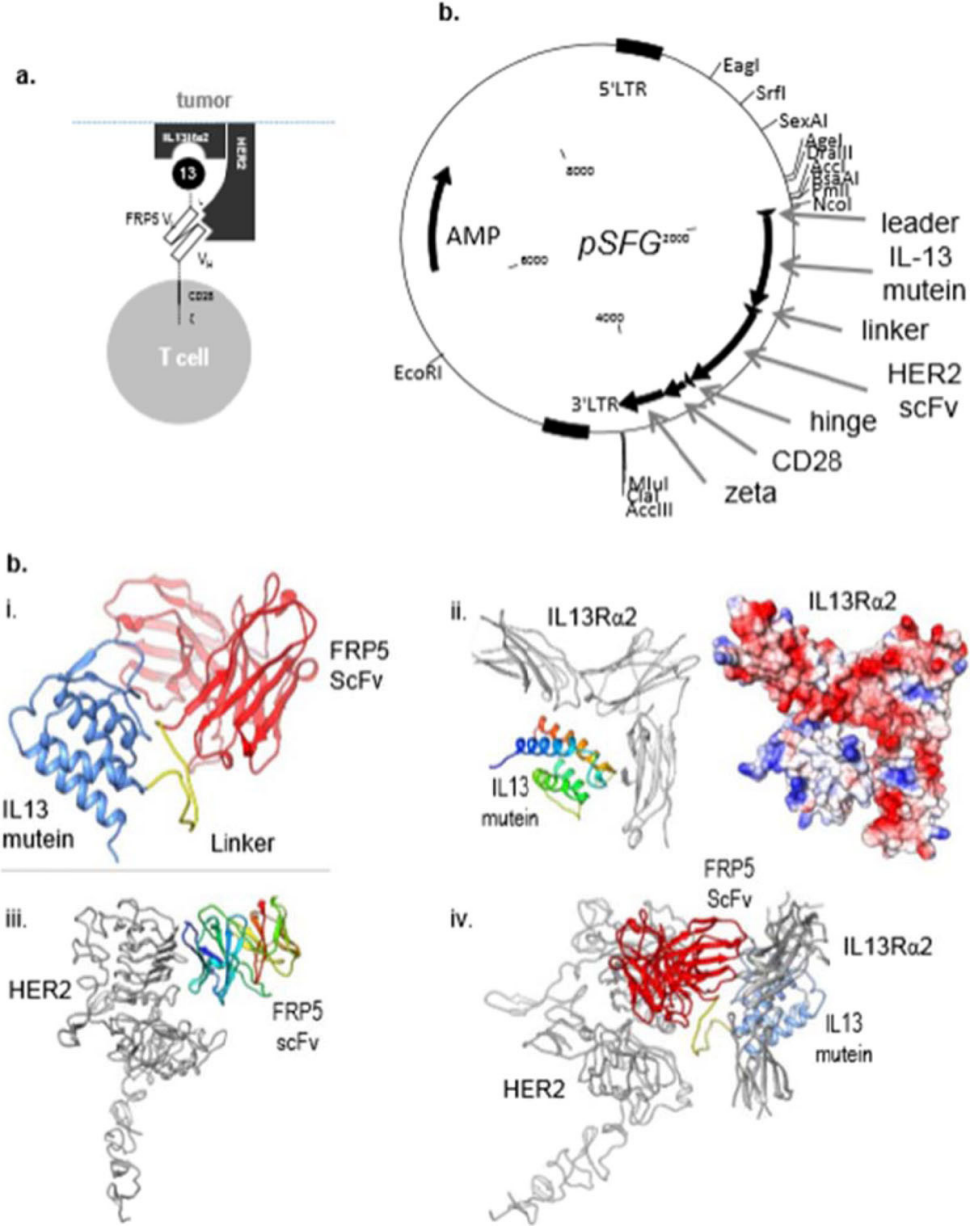


**Figure 2:**
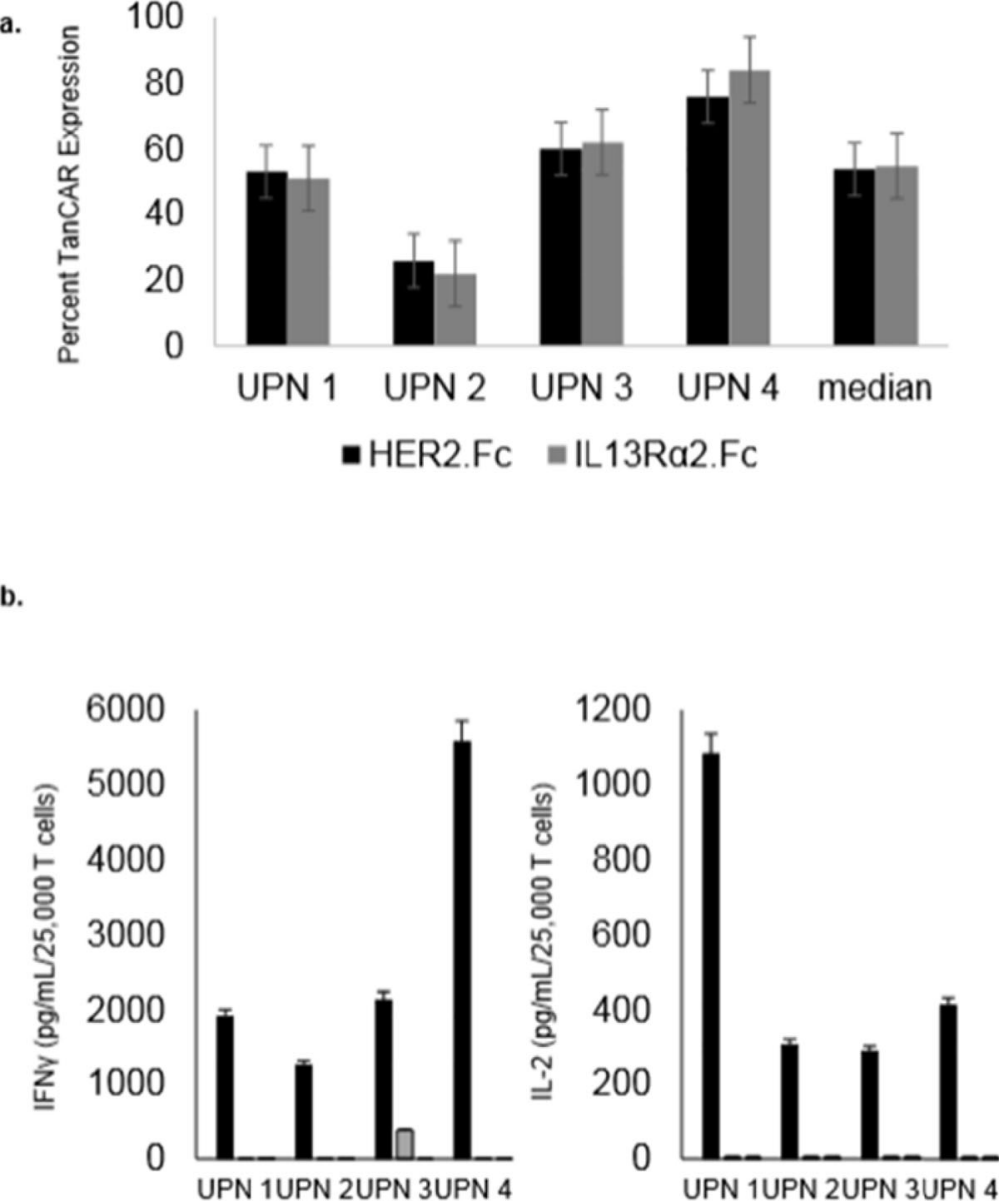


**Figure 3:**
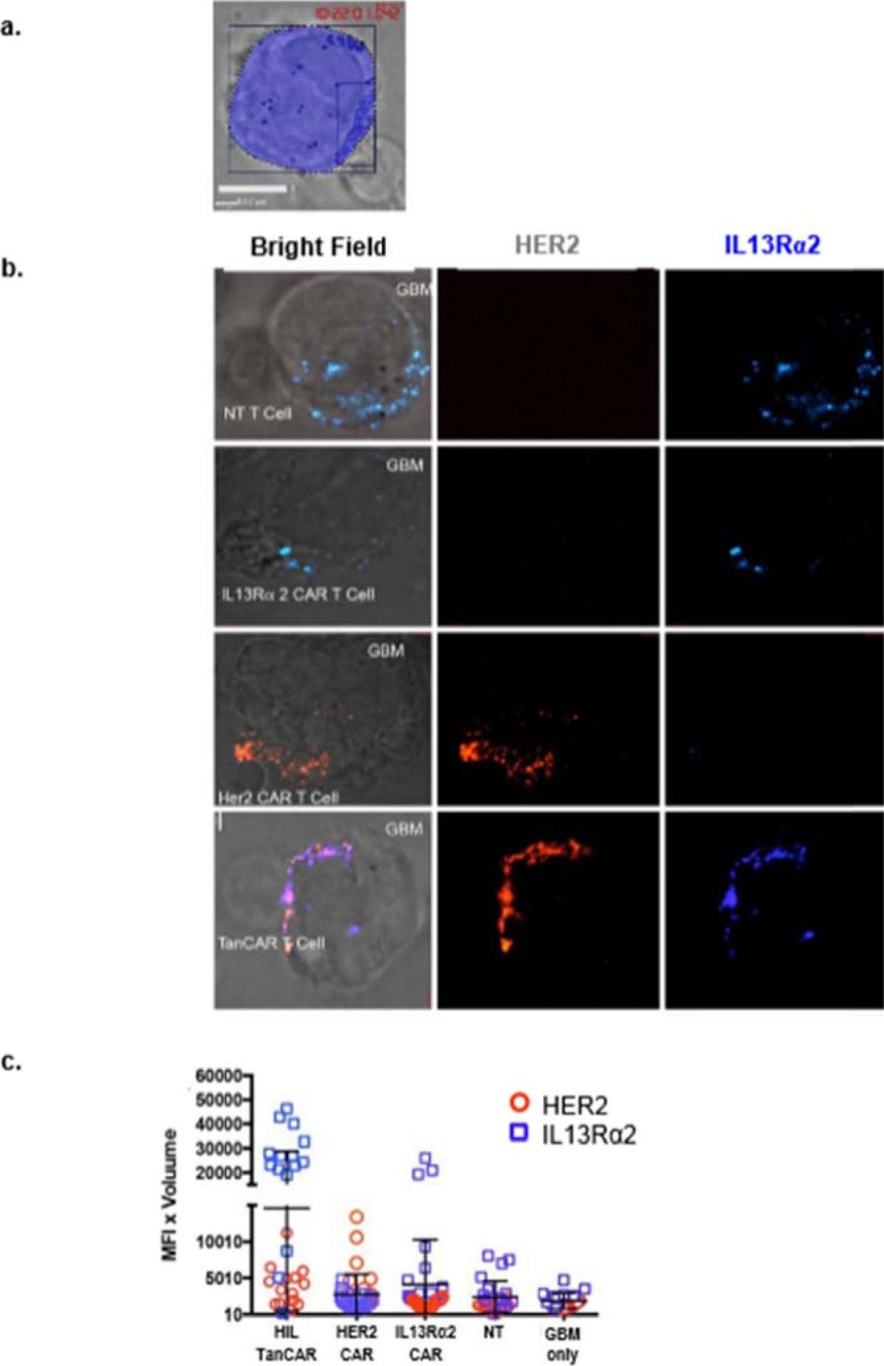


**Figure 4:**
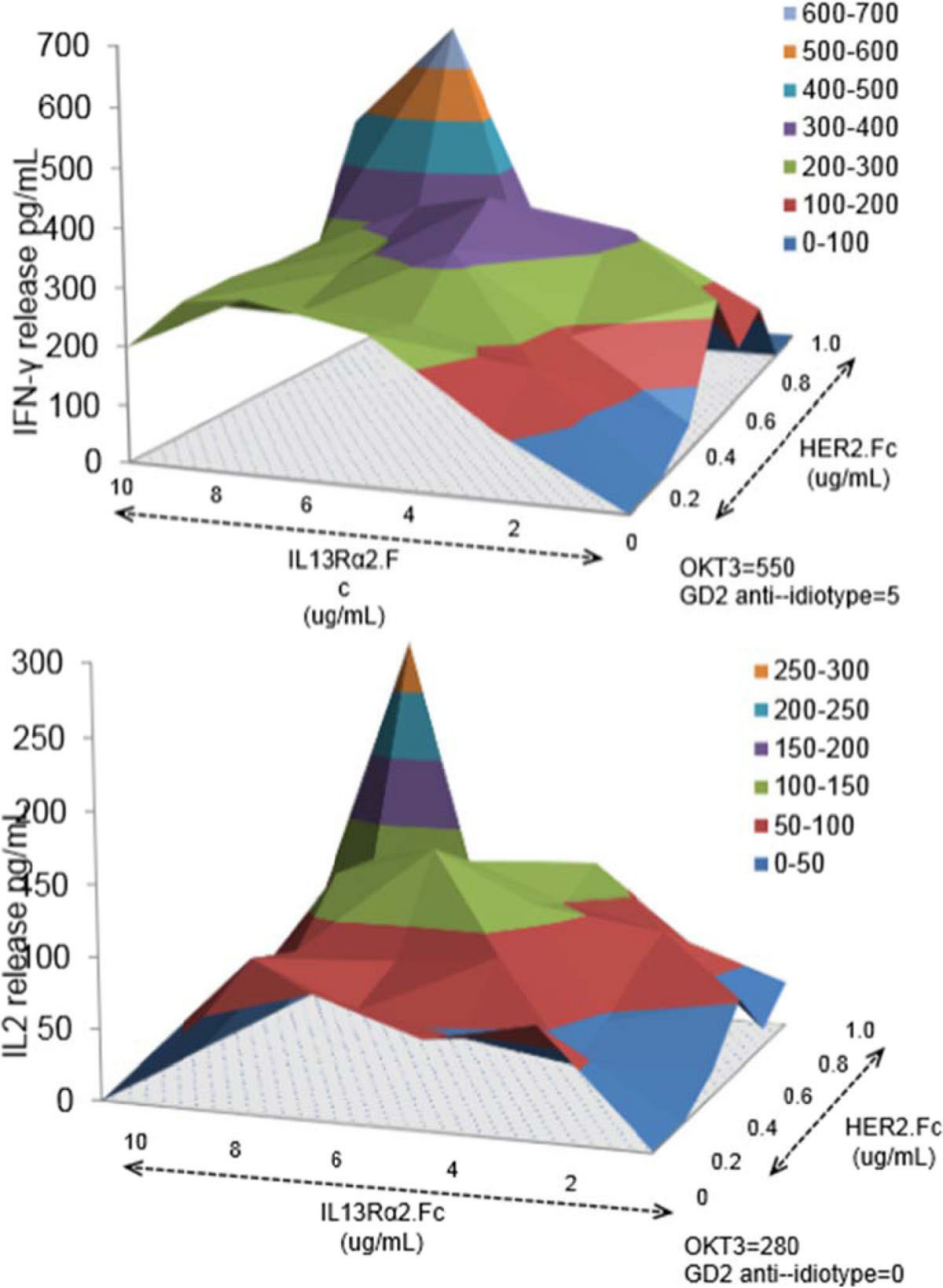


**Figure 5:**
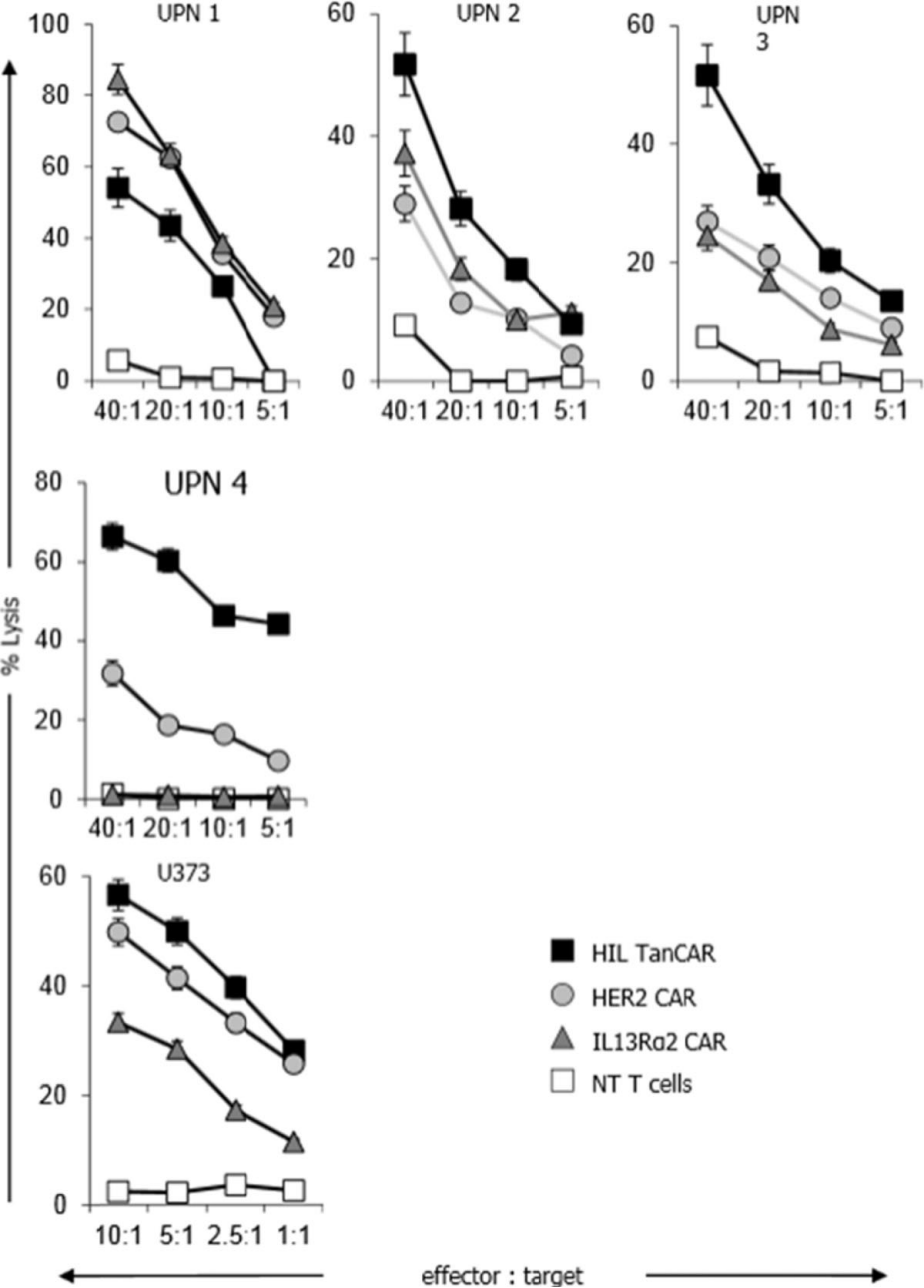


**Figure 6:**
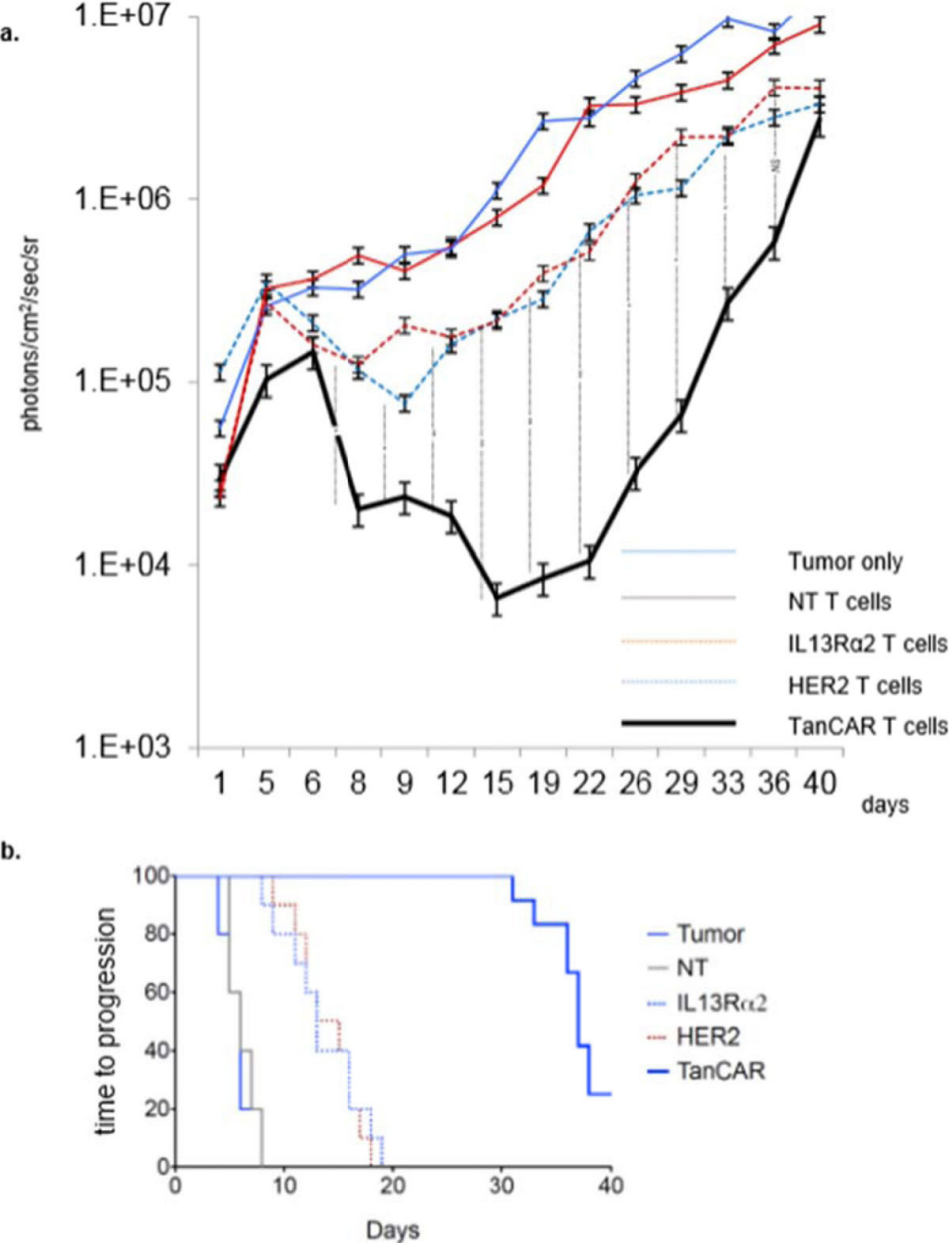


**Figure 7:**